# Near-Infrared Hyperspectral Classification of Waste Textiles Using Complementary Spectral Representations and a Convolution-Enhanced Patch Transformer

**DOI:** 10.3390/s26196069

**Published:** 2026-09-25

**Authors:** Xin Ru, Xingyu Chen, Laihu Peng, Chang Xuan, Yi Xu, Changjiang Wan

**Affiliations:** School of Mechanical Engineering, Xiasha Campus, Zhejiang Sci-Tech University, Hangzhou 310018, China; ruxin@zstu.edu.cn (X.R.); 2024220503012@mails.zstu.edu.cn (X.C.); laihup@zstu.edu.cn (L.P.); 202210501020@mails.zstu.edu.cn (C.X.); xuyi_117@163.com (Y.X.)

**Keywords:** near-infrared hyperspectral imaging, waste textiles, complementary spectral representations, fiber composition, convolution-enhanced Patch Transformer, interpretable deep learning

## Abstract

High-value recycling of waste textiles requires accurate fiber-composition identification, yet similar blends often exhibit overlapping near-infrared absorption bands and substantial within-class variation. Using 291 waste-textile swatches, this study develops ConvPatchTST, a convolution-enhanced Patch Transformer that integrates Raw and SNV (SG) spectra as complementary input channels. Overlapping convolutional patch embedding captures local continuous absorption features, while a Transformer encoder models cross-band dependencies. Spectral bands spanning 958.5–1646.6 nm were selected for model training and analysis. Under a single fabric-swatch-grouped internal train-validation split with pixel-level evaluation, ConvPatchTST achieves an accuracy of 0.966 and a macro F1 of 0.970, outperforming several baseline models. The Raw + SNV (SG) representation also surpasses single-channel, three-channel, and alternative dual-channel inputs. Pairwise analysis identifies statistically discriminative and highly attributed intervals for four similar material pairs. For polyester/polysp, the intervals near 1369.0–1379.6 nm show clear statistical-model agreement. These results indicate that complementary spectral representations and convolution-enhanced Patch modeling support accurate classification and provide band-level clues for interpreting model decisions. Generalization to external samples, batches, and instruments requires further validation.

## 1. Introduction

Waste textiles are a large-volume, compositionally complex solid waste stream that is difficult to recycle at high value within the textile supply chain. Fast-fashion consumption, shorter product replacement cycles, and growing textile production place persistent pressure on collection, reuse, and high-value circularity [1]. Compared with direct incineration, landfilling, or downcycling, accurate sorting by fiber composition is essential for closed-loop use of textile resources and directly affects recycled-fiber quality, the added value of recycled products, and the environmental benefits of recycling [2]. In practical recycling streams, fabrics differ simultaneously in fiber composition, fabric construction, and surface condition. Cotton, polyester, spandex, and blends with different compositions can appear similar, although their recycling routes and process compatibility differ substantially. A rapid, non-destructive, and robust identification method for industrial conveyor-belt settings is therefore fundamental to automated waste-textile sorting and is important for textile circularity, pollution and carbon reduction, and high-value material utilization.

Near-infrared spectroscopy characterizes overtone and combination bands associated with vibrations of chemical bonds such as O-H, C-H, and N-H. It supports rapid, non-destructive measurement and straightforward online integration, and has been used for identifying and sorting post-consumer textile waste [3]. Near-infrared hyperspectral imaging further integrates spatial and continuous spectral information, acquiring reflectance responses from different fabric locations within the same image and providing a data basis for pixel-level identification under complex surface conditions. Studies of industrial textile-waste sorting show that this technology identifies different fiber classes, although material mixtures, changes in surface condition, and spectral interpretation remain important barriers to practical application [4]. Deep-learning models, including convolutional neural networks, improve waste-textile recognition accuracy [5] and achieve strong classification performance on multiclass near-infrared data [6]. In spectral modeling, Savitzky–Golay smoothing, standard normal variate transformation, and partial least squares are used to suppress random noise, reduce scattering effects, and extract latent variables, respectively [7,8,9,10,11]. Hyperspectral imaging acquisition and analysis frameworks are also well established in food, agriculture, remote sensing, and industrial inspection [12,13]. For classification, PLS-DA constructs linear latent-variable discriminant models [14], RBF-SVM handles nonlinear class boundaries through kernel functions [15], and random forests provide robust nonlinear classification through tree ensembles [16]. Advances in deep feature extraction, three-dimensional and two-dimensional convolutional networks, and end-to-end hyperspectral classification continuously improve representations of local spectral shape and spatial–spectral coupling [17,18,19]. One-dimensional convolution and residual networks extract local patterns from continuous sequences and alleviate optimization difficulties in deep networks [20,21]. Transformers and Vision Transformers use self-attention to model long-range dependencies [22,23], while patch-based sequence models such as PatchTST offer a new perspective on learning relationships among continuous bands [24]. SpectralFormer further demonstrates the potential of Transformers for modeling spectral-sequence dependencies in hyperspectral classification [25]. Together, these methods support a comparison framework spanning conventional discriminant, convolutional, and attention-based models.

Despite this progress, reliable discrimination of similar materials in waste textiles and interpretation of their spectral mechanisms remain challenging. First, spandex content in cotton–spandex and polyester–spandex materials is generally low, whereas cotton–polyester blends are often classified using a threshold for cotton content. Differences between classes therefore appear more often as changes in the relative depth of broad absorption bands, shoulder positions, and local slopes than as separate characteristic peaks. Second, sample thickness, yarn arrangement, fabric structure, wrinkles, contamination, and local illumination can all alter reflectance levels, superimposing differences in material composition on non-target scattering variation. A single preprocessing method can suppress some interference but may also weaken absolute reflectance information with discriminative value. Third, existing studies commonly evaluate models using aggregate metrics such as Accuracy and F1-score. These metrics do not reveal the specific basis for separating similar material pairs or whether the model actually uses bands that are meaningful from a materials perspective. Compared with near-infrared spectroscopy, FTIR and Raman spectroscopy provide more direct evidence of material composition through molecular vibrations, functional-group responses, or characteristic scattering, and have been used to identify and characterize cellulosic and textile fibers [26,27,28]. Candidate intervals identified by near-infrared models should therefore be treated as classification and interpretation clues; their molecular-vibration assignments still require independent validation using FTIR, Raman spectroscopy, or related measurements.

Standardized mean differences express between-class mean differences relative to within-class variation [29,30]. Because material pairs do not have identical sample sizes and this study focuses on the relative strength of differences across bands, an Equal-Weighted Standardized Difference (EWSD) is constructed using an equal-weight average of the variances of the two groups. EWSD is a descriptive metric defined for the present spectral-analysis task and is not equivalent to Cohen’s d or Hedges’ g. In parallel, single-band AUC strength derived from ROC-AUC evaluates the ranking-based discriminative ability of an individual band for two classes [31], whereas Integrated Gradients quantifies the contribution of input features to model outputs from the perspective of model decisions [32]. Joint use of EWSD, single-band AUC strength, and Integrated Gradients provides complementary interpretive evidence from three levels: spectral statistical differences, single-band discriminative ability, and actual model use. This approach avoids interpreting the model solely from overall predictive performance or a single attribution result. Previous work also indicates that post hoc interpretation of black-box models requires careful assessment in light of the specific task, model mechanism, and domain knowledge [33,34].

This study addresses three questions: whether Raw and SNV (SG) channels provide complementary information when similar blends have overlapping bands and within-class variation; whether a convolution-enhanced Patch architecture jointly models local continuous spectral shape and cross-band relationships better than conventional convolutional models, Transformers, PatchTST, 1D-ResNet, SpectralFormer, and conventional classifiers; and to what extent statistically discriminative intervals agree with highly attributed model intervals. We construct ConvPatchTST for six waste-textile classes and conveyor-belt background data and compare it with PLS-DA, RBF-SVM, random forest, 1D-ResNet, and SpectralFormer under common data sources, preprocessing, swatch-grouped splits, and pixel-level evaluation. We test the hypotheses that complementary dual channels retain both absolute reflectance responses and stable spectral-shape information, that convolution-enhanced Patch encoding combined with Transformer encoding improves representations of weak local differences and blend-ratio changes, and that combining statistical discrimination with model attribution offers more cautious interpretive evidence than relying only on aggregate performance metrics.

## 2. Materials and Methods

### 2.1. Samples, Fiber Composition, and Hyperspectral Acquisition

Quantitative fabric samples supplied by the Zhejiang Institute of Quality Inspection (Hangzhou, China) were used. The dataset comprised six classes: pure cotton (cotton), pure polyester (polyester), cotton–spandex blends (cotsp), polyester–spandex blends (polysp), cotton–polyester blends with cotton contents of at least 35% (cotpoly35a), and cotton–polyester blends with cotton contents below 35% (cotpoly35b). The spandex content of both cotsp and polysp samples ranged from 1% to 10%. In total, 291 waste-textile swatches were collected. Their composition definitions and sample counts are listed in Table 1. An additional 20 sets of conveyor-belt background data (belt) were collected to improve recognition of non-textile background regions and reduce false detections in practical online sorting. The fabric samples used in this study were all undyed materials; therefore, the method proposed in this paper and the conclusions regarding its performance are primarily applicable to the identification of the fiber composition of undyed waste fabrics. Dyes and the additional spectral changes they may cause were not included in the scope of the current experiments.

Hyperspectral data are acquired using an HX17 near-infrared hyperspectral imaging system. The system uses push-broom line scanning to collect continuous spectra at different spatial locations on each sample. It consists primarily of a near-infrared hyperspectral camera, dual-side halogen light sources, a conveyor belt, and a drive-control unit. Its overall configuration is shown in Figure 1. During acquisition, samples are laid flat on the conveyor belt and scanned under stable, uniform halogen illumination. Standard white and black reference panels are used for reflectance calibration, yielding hyperspectral data cubes that contain both spatial and spectral information. The camera has a nominal near-infrared range of approximately 900–1700 nm, a spectral resolution of 8 nm, and a spatial resolution of 1280 × 1024 pixels. Because sensor response, source energy distribution, and reflectance-calibration error are more pronounced at the spectral ends, low-signal-to-noise channels at both ends are removed. The final dataset retains 202 valid channels spanning 958.5–1646.6 nm.

### 2.2. Spectral Preprocessing and Complementary Dual-Channel Representation

Raw spectra obtained by near-infrared hyperspectral imaging contain useful information about the chemical composition of textile materials, but they also inevitably include interference from instrument noise, sample surface condition, thickness variation, light scattering, illumination perturbations, and differences in fiber arrangement. These non-target factors cause spectral variation among samples of the same material and reduce effective separation between classes. Appropriate preprocessing is therefore applied before deep-learning classification while preserving complementary information that reflects differences in material composition and structure.

Raw spectra are first smoothed using Savitzky–Golay (SG) filtering with a window length of 15 and a polynomial order of 3. SG smoothing fits local polynomials to smooth spectral curves, reducing random high-frequency noise while preserving peak positions, spectral-shape variation, and the main absorption-band features of the original spectra. The processed curves are more continuous and show less random fluctuation, providing a more stable spectral basis for subsequent scatter correction and feature extraction.

Waste textile materials remain susceptible during acquisition to differences in sample thickness, surface texture, fiber arrangement, and local illumination, which produce baseline drift and overall amplitude variation across samples. To reduce scattering effects and non-target signal variation, SG-smoothed spectra are further corrected using the Standard Normal Variate (SNV) transformation to obtain the SNV (SG) representation. SNV mean-centers and standardizes each individual spectrum, reducing overall reflectance differences caused by scattering and baseline shift. The resulting representation emphasizes relative spectral-shape variation, local fluctuations, and proportional relationships among absorption bands, improving the consistency of spectral features across samples.

Figure 2 illustrates the effect of different preprocessing methods on near-infrared spectra of textile materials. Figure 2a shows raw spectra, which retain actual material reflectance responses, absorption-band intensities, and some amplitude information associated with fiber composition and fabric structure, but also contain random fluctuations and within-class variation. Figure 2b shows SG-smoothed spectra, which are smoother, suppress high-frequency noise, and preserve the major spectral trends. Figure 2c shows SNV (SG) spectra, which provide more stable relative spectral-shape information after noise suppression and scattering correction and help the model attend to local spectral shape, shoulder changes, and absorption-band ratios. Given the complementarity of information preserved by the two representations, Raw and SNV (SG) are used as a common dual-channel input, ensuring a fair comparison among subsequent models.

### 2.3. ConvPatchTST Spectral Classification Model

Near-infrared spectra are inherently one-dimensional continuous sequences with strong correlations between neighboring bands, and the shapes of local absorption bands are important for material recognition. Relationships among different absorption regions can also be jointly discriminative. For example, a blended material may not be determined by a single narrow band but may require simultaneous comparison of relative changes near 1100 nm, within 1350–1450 nm, and above 1500 nm. Conventional one-dimensional convolutional networks identify local patterns effectively but have limited ability to model coordination among distant bands. Standard Transformers capture global relationships, but direct processing of raw spectra may insufficiently emphasize local continuous spectral shapes. Conventional PatchTST models use patch partitioning and Transformer encoders for long-sequence modeling, yet direct patch partitioning can weaken local continuous spectral-shape information in near-infrared spectra.

This study develops ConvPatchTST. The model first receives Raw and SNV (SG) spectral channels. It then applies one-dimensional convolutional patch embedding with a kernel size of 16 and a stride of 4, producing 75% overlap between adjacent patches. This overlap allows the same local absorption band to be encoded by multiple receptive fields and avoids loss of peak, shoulder, or slope information at patch boundaries caused by rigid partitioning. After convolution generates patch tokens, the Transformer encoder uses self-attention to learn relationships among patches and constructs a joint representation of local spectral shapes and cross-interval dependencies. Finally, a classification head outputs predictions for seven classes.

As shown in Figure 3, the model contains five modules: dual-channel input, overlapping convolutional patch embedding, positional encoding, a Transformer encoder, and a classification head. The convolutional module models continuous absorption bands, whereas the Transformer module jointly models relationships across spectral bands.

To ensure reproducibility, the ConvPatchTST results reported in this paper were obtained using a single internal training/validation split grouped by blocks, with a random seed set to 43. The model employs overlapping convolutional patch embeddings with a patch size of 16 and a stride of 4, generating 47 spectral patches from 202 valid bands, followed by a CLS token. Validation is performed at the end of each epoch, and the checkpoint with the highest validation macro F1-score is retained. The main architecture and training parameters (including Transformer depth, number of attention heads, hidden layer dimensions, embedding dimensions, and early stopping criteria) are listed in Table 2.

### 2.4. Paired Spectral Discrimination and Model Interpretability

#### 2.4.1. Paired Material Combinations

To prevent mixed analysis of all seven material classes from masking subtle spectral differences between similar classes, four material pairs are selected: cotton/cotsp, polyester/polysp, cotton/cotpoly35a, and cotpoly35a/cotpoly35b. The cotton/cotsp pair examines spectral changes induced by adding 1–10% spandex to a cotton-fiber system, while polyester/polysp examines the effect of low-proportion spandex on spectral shape in a polyester matrix. In both comparisons, spandex content is low and the overall spectrum remains dominated by cotton or polyester, making these pairs suitable for assessing the model’s ability to recognize weak local differences.

The cotton/cotpoly35a pair analyzes differences between pure cotton and cotton–polyester blends with relatively high cotton content, focusing on the effects of added polyester on cellulose-related absorption bands and overall spectral shape. The cotpoly35a/cotpoly35b pair analyzes continuous spectral changes when cotton content crosses the 35% labeling threshold. The chemical compositions of this pair are not sharply separated, and discrimination depends more on the relative depth of broad absorption bands, shoulder features, and joint relationships among multiple bands. Together, the four pairs cover two typical cases, low-proportion spandex addition and changes in the cotton–polyester ratio, and support systematic evaluation of consistency between statistical discrimination and model attribution.

#### 2.4.2. Intrinsic Spectral Evidence for Discrimination

For each material pair, the mean spectrum and standard deviation of each class are first calculated at every band to characterize between-class central tendency and within-class variation. On this basis, the Equal-Weighted Standardized Difference (EWSD) quantifies the strength of between-class differences at each band. Let the mean reflectances of materials A and B at band i be denoted by $\mu_{\left\{A,i\right\}}$ and $\mu_{\left\{B,i\right\}}$, and let their standard deviations be denoted by $s_{\left\{A,i\right\}}$ and $s_{\left\{B,i\right\}}$ EWSD is defined as

(1) $${EWSD}_{i}=\frac{\left|\mu_{\left\{A,i\right\}}-\mu_{\left\{B,i\right\}}\right|}{\sqrt{\frac{s_{\left\{A,i\right\}}^{\left\{2\right\}}+s_{\left\{B,i\right\}}^{\left\{2\right\}}}{2}}}$$

This metric considers both the mean difference between the two materials and their within-class dispersion. For waste textiles, fabric thickness, yarn arrangement, surface fuzz, and scattering conditions can cause spectral variation among samples of the same class. Comparing only the distance between two mean spectra therefore cannot identify discriminative bands reliably. A larger EWSD value indicates that the between-class difference at that band is more stable relative to within-class variation and can serve as the first level of evidence for material spectral differences. EWSD uses an equal-weight average of the two variances and is intended to compare the relative strength of differences across bands rather than infer a population effect size from sample sizes. For a candidate discriminative interval R containing continuous bands, the mean EWSD is the arithmetic average of EWSD values within R. It summarizes the overall statistical discrimination of a continuous interval and avoids judging its importance from one maximal band alone.

Single-band AUC strength is also calculated to evaluate the ranking-based discriminative ability of each individual band for the two materials. Specifically, band i alone is treated as a one-dimensional feature, and its ROC-AUC for separating the two classes is calculated. An AUC close to 1 or 0 indicates strong ranking-based discrimination, with the two values differing only in direction. To remove the effect of direction, AUC is converted into a direction-invariant strength metric:

(2) $$S_{i}=2\times \max\left(AUC_{i},1-AUC_{i}\right)-1$$

This metric ranges from 0 to 1. A value close to 0 indicates weak ranking-based discrimination at that band, whereas a value close to 1 indicates that the band alone provides strong ranking-based discrimination. Unlike EWSD, single-band AUC strength does not depend on the specific form of the mean and variance and more directly reflects the ability of an individual band to rank samples from two materials. The two metrics therefore provide complementary information from standardized mean differences and sample-ranking relationships.

Candidate discriminative intervals are not defined from isolated peaks at single wavelengths. Instead, EWSD and single-band AUC strength are combined into a band-discrimination score, and continuous high-scoring regions are extracted. Continuous intervals are used rather than point peaks because near-infrared absorption commonly arises from overtones and combination bands of O-H, C-H, and N-H vibrations and therefore appears as broad, overlapping absorption bands. An isolated sharp peak may be affected by noise, local scattering, or anomalous samples, whereas multiple adjacent bands with high EWSD and AUC strength are more likely to reflect stable material spectral differences. For material pairs with different sample counts, these metrics are used as descriptive tools to compare relative discriminative ability across bands.

#### 2.4.3. Model Attribution Evidence

Statistical differences do not necessarily imply that the model uses those differences. To identify the spectral regions that ConvPatchTST actually relies on when discriminating paired materials, Integrated Gradients is used for model attribution. Let the model-output logit be f(x), the input spectrum be x, and the baseline spectrum be x′. The Integrated Gradients value of band i is defined as

(3) $$IG_{i}=\left(x_{i}-x_{i}^{\prime }\right)\int_{0}^{1}\frac{\partial f\left(x^{\prime }+\alpha \left(x-x^{\prime }\right)\right)}{\partial x_{i}d\alpha }$$

The attribution target was defined as the paired logit difference. Absolute attribution means are then calculated for the two input channels for each material class and combined using an equal-weight average to obtain Mean|Integrated Gradients|. This aggregation reduces the effect of unequal sample sizes on attribution results. A larger value indicates that variation at the corresponding band has a greater effect on the model output used to separate the material pair. For each extracted high-attribution interval, the mean attribution value is the arithmetic average of Mean|Integrated Gradients| across the interval. It summarizes the model’s overall reliance on a continuous interval and avoids misinterpreting an accidental single-band attribution peak as stable model attention.

This procedure yields two complementary forms of evidence. EWSD and single-band AUC strength evaluate whether the spectral data themselves contain regions with relative discriminative ability, whereas Mean|Integrated Gradients| evaluates whether the trained ConvPatchTST actually uses these regions. When statistically discriminative intervals overlap with high-attribution intervals, the region contains both material spectral differences and a substantial contribution to model decisions and can be regarded as a relatively reliable interpretable discriminative region. When the two types of intervals do not fully overlap, the model may instead discriminate using multiple local regions and their cross-interval combinations.

### 2.5. Experimental Design and Evaluation Metrics

Neighboring pixels from the same fabric piece in a hyperspectral image produce many similar spectra. Under pixel-level random splitting, pixels from the same fabric piece can enter both the training and validation sets, inflating validation metrics. To avoid this issue, we use a fabric-swatch-grouped split: each swatch is an independent splitting unit and never appears in both training and validation sets. Each fabric piece serves as an independent splitting unit, all pixel-level spectra from the same sample are assigned to the same data subset, and the data are randomly divided into training and validation sets at an approximate ratio of 8:2. The split is shown in Table 3. All reported performance values are obtained from pixel-level validation data under this single internal train-validation split.

Model performance is evaluated using accuracy, precision, recall, and macro F1. Accuracy reflects the overall proportion of correct predictions. Precision measures the proportion of correct samples among those predicted as a given class. Recall reflects the completeness with which samples of a true class are identified. Macro F1 assigns equal weight to each class and is suitable when class pixel counts are not fully balanced. Experiments also compare different input channels, dual-channel preprocessing combinations, and CNN, Transformer, PatchTST, SpectralFormer, ConvPatchTST, and random forest models to validate dual-channel complementarity and the proposed architecture.

## 3. Results and Discussion

### 3.1. Overall Spectral Characteristics of Textile Materials

Across 958.5–1646.6 nm, the mean spectra of the six textile materials and conveyor-belt background vary continuously, with different degrees of spectral-shape overlap among textile materials. A single band is therefore insufficient for stable discrimination, and mean curves do not represent the complete range of within-class variation. Raw spectra retain differences in reflectance level and absorption intensity but also include variation from fabric thickness, fiber arrangement, surface texture, and scattering conditions. SG smoothing mainly suppresses high-frequency noise, while SNV (SG) further reduces baseline and global amplitude variation and highlights relative local slopes, shoulder features, and broad-band shapes. These observations indicate that material discrimination in this dataset relies more on the relative shapes of multiple continuous bands and their combinations than on an isolated characteristic peak.

In terms of composition, cotton and polyester represent the dominant spectral characteristics of cellulose and polyester matrices, respectively. Under the near-infrared range and blend ratios used here, the 1–10% elastane content in cotsp and polysp is more likely reflected in changes in local intensity, slope, and shoulder features of broad absorption bands dominated by the main fiber, rather than in an independent stable elastane peak. The cotpoly35a and cotpoly35b labels are defined by whether cotton content reaches 35%, and their difference mainly appears as broad-band shape variation caused by changing relative contributions of cotton and polyester absorption. Mean spectra are therefore used only to describe class central trends and spectral overlap; candidate discriminative intervals and bands that the model actually attends to are jointly identified below using EWSD, single-band AUC strength, and Integrated Gradients.

### 3.2. Main Discriminative Spectral Intervals for Four Pairs of Similar Materials

To explain why similar materials are separable from their spectra, Figure 4 presents mean spectra, EWSD, single-band AUC strength, and continuous highly discriminative regions indicated by yellow shading for all four material pairs. The upper mean spectra show overall shape differences and within-class variation. The EWSD curves measure stable standardized differences, and the single-band AUC strength curve measures the ranking-based discriminative ability of an individual band and is abbreviated as AUC in Figure 4. Shaded regions indicate intervals that have high combined EWSD and AUC strength and occur continuously across adjacent bands. Compared with mean spectra alone, this combination distinguishes apparent mean differences from regions with stable statistical discriminative ability. Results for the four material pairs are shown in Figure 4 and Table 4.

For cotton/cotsp, Figure 4a and Table 4 show that statistically discriminative intervals are mainly located at 1362.1–1407.1 nm, 961.6–971.8 nm, and 1150.9–1157.9 nm, with mean EWSD values of 0.469, 0.456, and 0.457, respectively. Because elastane content in cotsp is low, the overall spectrum remains dominated by cotton. Elastane is more likely to alter the relative depth and shape of local broad bands. These intervals should therefore be regarded as candidate discriminative regions formed jointly by the cotton matrix, small amounts of elastane, and textile scattering, rather than as independent elastane peaks. Their dispersed distribution indicates bandwise heterogeneity in differences caused by a low-proportion component. Subsequent analysis should focus on relative spectral shape, local slope, and within-class stability within continuous intervals. Establishing a quantitative relationship between discriminative intervals and elastane content would require standard samples with matched cotton content and controlled elastane ratios.

For polyester/polysp, Figure 4b and Table 4 identify statistically discriminative intervals at 958.5–985.5 nm, 1369.0–1379.6 nm, and 1133.5–1154.4 nm, with mean EWSD values of 0.449, 0.408, and 0.395, respectively. Compared with cotton/cotsp, the differences caused by small amounts of elastane in the polyester matrix are weaker overall, but several continuous bands still show repeatable statistical characteristics. The 1369.0–1379.6 nm interval strongly corresponds to the subsequent model-attribution interval and provides the most representative statistical evidence for this material pair. Reliable classification does not require significant differences at every wavelength; several stable intervals can provide complementary information for identification through joint spectral shape. The statistical difference near 1369.0–1379.6 nm consequently has strong interpretive value for industrial sorting.

For cotton/cotpoly35a, Figure 4c and Table 4 show that statistically discriminative intervals are concentrated at 1500.5–1567.0 nm, covering 21 continuous bands with a mean EWSD of 1.214, substantially higher than for the other material pairs. This finding indicates that the difference between cotton-rich cotton–polyester blends and pure cotton is mainly expressed as an overall shape change in a long-wavelength broad absorption band. For cotpoly35a/cotpoly35b, Figure 4d and Table 4 identify discriminative intervals at 1126.7–1175.8 nm and 1493.9–1510.5 nm, indicating that crossing the 35% cotton-content labeling threshold produces differences in continuous spectral shapes in both middle and long wavelength regions. Because the boundary is defined by whether cotton content reaches 35%, this pair tests the response of the model to blend-ratio variation. The continuous intervals rather than isolated peaks suggest that ratio variation primarily affects the relative depth, shoulders, and local slopes of broad absorption bands. This also supports convolutional Patch extraction of continuous spectral shape followed by Transformer modeling of joint relationships among intervals.

### 3.3. Band Attribution and Spectral Mechanisms of ConvPatchTST

Although the combined EWSD and single-band AUC strength curves identify statistically high-scoring regions, they do not establish that the model relies on those regions. To examine bands actually used by ConvPatchTST, Figure 5 presents Mean |Integrated Gradients| attribution curves for the four material pairs and marks the high-attribution intervals and mean attribution values reported in Table 4. A higher attribution value indicates a greater influence of the corresponding band and local variation on the pairwise logit difference.

For cotton/cotsp, Figure 5a and Table 4 show high-attribution intervals at 1623.7–1630.6 nm, 1590.6–1607.0 nm, and 1338.8–1345.4 nm, with mean attribution values of 0.519, 0.480, and 0.364, respectively. Compared with the statistically discriminative intervals, model attribution is distributed across several local regions. This indicates that discriminating cotton from cotton–elastane relies more on multiple regional spectral shapes and their combination, consistent with weak differences caused by low-proportion elastane and pronounced overlap in main-fiber absorption bands.

For polyester/polysp, Figure 5b and Table 4 show a continuous high-attribution interval from 1372.4 to 1413.6 nm with a mean attribution value of 0.945, covering the statistically discriminative interval at 1369.0–1379.6 nm. This overlap is the most direct evidence of statistical-model agreement among the four pairs and shows that the model effectively uses local spectral-shape differences between polyester and polyester–elastane in this region. The region may cautiously be related to C-H-associated vibrations of polyester and elastane and their overlapping effects, but it should not be assigned uniquely to one functional group.

For cotton/cotpoly35a, Figure 5c and Table 4 show that the main high-attribution interval is 1620.4–1630.6 nm with a mean attribution value of 0.541, which does not overlap the statistically discriminative interval at 1500.5–1567.0 nm. The model may therefore use local spectral shape in longer-wavelength regions and its joint changes with other bands to discriminate the pair. A statistically high-EWSD region can reflect material differences without being the model’s sole decision basis.

For cotpoly35a/cotpoly35b, Figure 5d and Table 4 show high-attribution intervals at 1335.3–1345.4 nm, 1297.3–1304.3 nm, and 1623.7–1630.6 nm, with mean attribution values of 0.515, 0.433, and 0.348. Compared with the statistical intervals at 1126.7–1175.8 nm and 1493.9–1510.5 nm, attribution is distributed across different intervals, indicating that the cotton-content threshold difference is represented mainly through multiple local regions and their relative spectral shapes.

Overall, the four material pairs show distinct statistical–attribution relationships. Cotton/cotsp exhibits weak multi-region differences caused by low-proportion elastane; polyester/polysp shows the clearest statistical–attribution correspondence at 1372.4–1413.6 nm; and cotton/cotpoly35a and cotpoly35a/cotpoly35b exhibit cross-band combinatorial modeling. The investigated range spans 958.5–1646.6 nm, within which absorption responses from cotton fibers, polyester, and elastane overlap across several broad regions. In particular, for cotsp and polysp with 1–10% elastane, elastane is more likely reflected in the relative intensity, peak width, and local slopes of broad bands dominated by the main fiber than in a stable elastane-specific peak.

Accordingly, these intervals are interpreted as candidate regions that are statistically discriminative and assigned relatively high decision contributions by ConvPatchTST, rather than being uniquely attributed to any single chemical bond. The present results support their relevance to material differences and model decisions, but specific molecular-vibration assignments require further validation through FTIR, Raman spectroscopy, peak-width analysis, and controlled-composition experiments.

### 3.4. Validation of Complementary Dual-Channel Spectral Representations

Raw + SNV (SG) achieved high F1-scores for most material classes, including 0.996, 0.994, 0.931, and 0.960 for polyester, polysp, cotpoly35a, and cotpoly35b, respectively. Relative to the Raw single channel, these values improved by 0.022, 0.063, 0.034, and 0.100, respectively. The dual-channel F1-scores for cotton and cotsp were 0.945 and 0.961, respectively; the cotton score exceeded that of the Raw channel (0.918), whereas Raw performed slightly better for cotsp (0.974). These results indicate that raw reflectance contains useful information about material-response intensity, but a single Raw channel can miss subtle discriminative cues associated with blend-ratio variation and low-proportion elastane addition. Although the SG and SNV (SG) single channels help suppress noise and scattering, they performed worse than the dual channel for several classes, suggesting that preprocessing can also weaken raw amplitude information related to material composition.

As shown in Table 5, Raw + SNV (SG) provided the clearest improvement for several blended materials. The F1-score for polysp increased from 0.931 with Raw alone to 0.994, that for cotpoly35a increased from 0.897 to 0.931, and that for cotpoly35b increased from 0.860 to 0.960, yielding gains of 0.063, 0.034, and 0.100, respectively. Cotpoly35a and cotpoly35b are both cotton–polyester blends whose class boundary is determined primarily by cotton content; relying solely on raw reflectance intensity or corrected spectral shape may therefore miss some discriminative cues. The dual channel supplies both absolute reflectance responses and relative spectral-shape information, helping the model extract more stable combined features under different sample conditions.

Notably, the Raw + SG + SNV (SG) three-channel input does not consistently outperform Raw + SNV (SG). It yields a higher F1-score only for cotsp, while its F1-scores for polyester, polysp, cotpoly35a, and cotpoly35b are lower than those of the dual-channel representation. Performance gains are therefore not produced simply by adding channels, but depend on complementarity among channels. The SG channel substantially overlaps in information content with Raw and SNV (SG); an additional channel may introduce redundancy and increase the learning burden. The value of the dual channel thus lies not in increasing the number of spectra, but in retaining two physically distinct types of information with few channels: raw reflectance responses and stable spectral shape after correction (see Figure 6).

To further validate preprocessing combinations, the dual-channel input form is fixed and four preprocessing schemes are compared. The designs are listed in Table 6 and the results are shown in Figure 7 and Table 7. The simple mean of classwise F1-scores across seven classes is 0.970 for Scheme A, exceeding 0.928, 0.932, and 0.924 for Schemes B, C, and D, respectively. Scheme A also achieves the highest F1-score among the four schemes for polyester, cotsp, polysp, and cotpoly35b, indicating stronger stability in overall performance and across most key classes.

As shown in Table 7, Scheme A maintains the highest F1-score for most key classes. Schemes B and C perform better for individual classes but are less stable. Scheme B may retain unsmoothed random fluctuations, Scheme C uses channels with similar sources, and Scheme D lacks raw reflectance information. Their overall performance is therefore lower than that of Raw + SNV (SG).

### 3.5. A Comparison of the Classification Performance of ConvPatchTST

To ensure a fair comparison, all models were evaluated using the same data sources, class-filtering rules, effective spectral range, input channels, and patch-grouping and data-partitioning strategy. Deep-learning models were trained for a common maximum number of epochs with early stopping, whereas conventional models were fitted using the same training and validation sets.

Figure 8 and Table 8 compare CNN, Transformer, PatchTST, and ConvPatchTST. Based on the classwise F1-scores in Table 8, ConvPatchTST has a simple mean F1-score of 0.970 across the seven classes, exceeding 0.932 for Transformer, 0.928 for PatchTST, and 0.888 for CNN. Except for belt, ConvPatchTST achieves the highest F1-score among the four models for all six textile classes. Under the present split and validation conditions, the convolution-enhanced Patch architecture effectively combines local continuous spectral-shape information with cross-band dependencies and thereby improves near-infrared spectral classification.

As shown in Table 8, all models achieve strong recognition performance for pure-fiber materials, while performance differences are concentrated in blended materials. ConvPatchTST obtains F1-scores of 0.996, 0.994, 0.960, and 1.000 for polyester, polysp, cotpoly35b, and belt, respectively, indicating stable recognition of pure polyester, polyester–elastane, low-cotton cotton–polyester blends, and conveyor-belt background. F1-scores of 0.945 and 0.961 for cotton and cotsp show that complete multi-band information and its combined relationships provide effective discriminative evidence despite the spectral similarity of the two classes. The F1-score for cotpoly35a is 0.931, higher than the CNN, Transformer, and PatchTST values of 0.870, 0.911, and 0.890, but it remains relatively low among ConvPatchTST textile classes. This may reflect its location in a continuous transition range between pure cotton, cotton–elastane, and cotton–polyester blends with different ratios, together with sensitivity to textile construction and surface condition.

Using the simple mean of classwise F1-scores in Table 8, ConvPatchTST improves over 1D-CNN, Transformer, and PatchTST by 8.1, 3.7, and 4.1 percentage points, respectively. Its advantage arises from complementary Raw and SNV (SG) representations, convolutional extraction of local spectral shape, and Patch-Transformer modeling of cross-band relationships, rather than from a single band or module. ConvPatchTST achieves higher F1-scores than all three comparison models for each of the six textile classes. This finding is consistent with the validation of Raw + SNV (SG) in Section 3.4: raw reflectance retains material-response intensity, SNV (SG) enhances spectral shape and local variation, convolution extracts local shapes within continuous bands, and patch representations with Transformer encoding establish relationships among bands. The advantage of ConvPatchTST therefore reflects the combined effect of dual-channel spectral representation, local spectral-shape extraction, and cross-band relationship modeling.

### 3.6. Comparison of ConvPatchTST with PLS-DA, RBF-SVM, Random Forest, 1D-ResNet, and SpectralFormer

To ensure a fair comparison, ConvPatchTST, PLS-DA, RBF-SVM, Random Forest, 1D-ResNet, and SpectralFormer were all evaluated using the same Raw + SNV (SG) input representation, which comprised two channels: the raw spectrum and the spectrum subjected to SNV transformation followed by Savitzky–Golay smoothing. All models were evaluated using the same 80/20 training–validation split, with the data grouped by single-pass blocks, and the same standardization procedure, in which the standardization parameters were estimated exclusively from the training data and then applied to the corresponding validation data. No grid search, random search, or Bayesian hyperparameter optimization was conducted on the validation set. Instead, each method was evaluated using a fixed configuration specified before model evaluation. The neural network models used the same training budget and early-stopping strategy, where applicable, whereas PLS-DA, RBF-SVM, and Random Forest were each fitted once on each training partition because these non-iterative estimators do not involve training epochs or early-stopping mechanisms. Accordingly, the comparison preserved the model-specific configurations of each algorithm family while maintaining consistency in the input representation, data partitioning, normalization procedure, and hyperparameter-tuning strategy.

As shown in Figure 9, ConvPatchTST achieved the highest values among the evaluated models for accuracy (0.966), precision (0.969), recall (0.970), and macro F1-score (0.970). For 1D-ResNet, the accuracy and macro F1-score were 0.931 and 0.935, respectively. The macro F1-scores of SpectralFormer and RBF-SVM were both 0.921, compared with 0.880 for Random Forest and 0.705 for PLS-DA. Relative to 1D-ResNet, ConvPatchTST improved both accuracy and macro F1-score by 3.5 percentage points, indicating superior overall classification performance under the present evaluation setting.

As shown in Table 9, ConvPatchTST achieved F1-scores of 0.945, 0.996, 0.961, 0.994, 0.931, 0.960, and 1.000 for the seven material classes, respectively. With the exception of cotton, it obtained the highest F1-score among all models for the six textile classes, while its performance on belt was comparable to that of the other models. 1D-ResNet outperformed ConvPatchTST only for cotton; however, its performance on blended classes, including polysp, cotpoly35a, and cotpoly35b, was markedly lower. This pattern suggests that, although the residual architecture can effectively extract local spectral shapes, its capacity to model cross-band relationships associated with low-proportion components and variation in blend ratios remains limited. SpectralFormer captured long-range band relationships for some classes, but its F1-scores for polysp, cotpoly35a, and cotpoly35b were only 0.901, 0.910, and 0.834, respectively. RBF-SVM and Random Forest also performed less well than ConvPatchTST in recognizing continuous spectral-shape variations in blended materials. The F1-score of PLS-DA for cotsp was only 0.299, indicating that linear models have difficulty representing the subtle differences associated with low spandex proportions.

For the more difficult blended classes, the advantage of ConvPatchTST was not driven primarily by easily classified samples or the conveyor-belt background. All models achieved an F1-score of 1.000 for belt, whereas their differences were more evident for classes characterized by subtle compositional variation or cotton contents near the 35% labeling threshold. For cotsp, ConvPatchTST achieved an F1-score of 0.961, exceeding the scores of 0.946, 0.918, 0.899, 0.808, and 0.299 obtained by 1D-ResNet, SpectralFormer, RBF-SVM, Random Forest, and PLS-DA, respectively. For polysp and cot-poly35b, its F1-scores were 0.994 and 0.960, respectively, representing improvements of 8.4 and 13.8 percentage points over 1D-ResNet and 9.3 and 12.6 percentage points over SpectralFormer. These classes involve subtle spectral differences arising from low spandex proportions and broad spectral-shape changes associated with cotton content crossing the 35% threshold. Thus, the performance advantage of ConvPatchTST was most evident for blended materials that were more difficult to sort.

Overall, local feature extraction alone or global relationship modeling alone was insufficient to robustly address the complex spectra examined in this study. ConvPatchTST uses overlapping convolutional patches to extract local spectral shapes from continuous bands and a Transformer to integrate cross-band information across patches. This design better represents subtle multi-region differences jointly associated with component proportions, scattering effects, and fabric states. Under the same sample partitioning, effective spectral range, and Raw + SNV (SG) dual-channel input conditions, ConvPatchTST achieved the best overall metrics and the highest performance for most textile classes. These findings support the interpretation that the combination of local spectral-shape extraction and cross-band relationship modeling improves classification performance. However, this conclusion is limited to the present sample source and a single internal validation protocol; it should not be directly extrapolated to external samples or independent instruments. Its practical generalizability requires further assessment using independent batches, diverse fabric states, and cross-device data.

## 4. Conclusions and Future Work

To address spectral overlap, subtle blend differences, and insufficient model interpretability in near-infrared hyperspectral recognition of waste textiles, this study constructs a Raw and SNV (SG) dual-channel spectral representation and a ConvPatchTST classification model. The Raw channel preserves absolute reflectance responses and absorption-band intensity information, whereas the SNV (SG) channel emphasizes stable spectral shapes after smoothing and scatter correction. Overlapping convolutional patch embedding extracts local continuous absorption features, and the Transformer encoder models cross-band relationships. After excluding low signal-to-noise channels at both spectral ends, the model is trained and analyzed using 202 effective bands from 958.5 to 1646.6 nm.

Under a single internal train–validation split grouped by fabric swatch, ConvPatchTST achieves pixel-level validation accuracy of 0.966, precision of 0.969, recall of 0.970, and macro F1 of 0.970. These results indicate strong classification performance under the current dataset and validation setting, but they cannot be directly generalized to external samples, different batches, or independent instruments. Results from single-channel and comparison models jointly show that improved performance is associated with the complementarity of information retained by Raw and SNV (SG), local spectral-shape extraction, and cross-band relationship modeling.

Pairwise interpretable analyses show that cotton/cotsp, polyester/polysp, cotton/cotpoly35a, and cotpoly35a/cotpoly35b all contain continuous or multi-region statistically discriminative intervals and highly attributed model intervals. For polyester/polysp, the statistically discriminative interval at 1369.0–1379.6 nm partially overlaps the highly attributed model interval at 1372.4–1413.6 nm, indicating that the model uses spectral-shape information related to class separation in this neighboring region. Attribution intervals are more dispersed for cotton–elastane and cotton–polyester-ratio-related materials, suggesting that the model jointly uses multiple local regions and their cross-band combinations rather than relying on a single continuous band.

This study has several limitations. First, cotsp and polysp are specified only as having elastane contents from 1% to 10%; exact ratios for individual samples are unavailable, so the relationships among fiber ratios, spectral-shape changes, and model outputs cannot be quantified further. Second, sample sources and batches are relatively limited, and stains, moisture, dyes, textile construction, thickness, and usage condition may affect generalization in industrial settings. It should also be noted that the current study primarily focused on validating the method using uncolored waste fabrics; therefore, the results of this paper cannot yet directly prove that the method is equally effective for dyed fabrics. Since dyes may alter a fabric’s overall reflectance, absorption intensity, and local spectral shape, extending this method to fabrics of different colors, dye types, and degrees of dyeing represents a promising direction for future research. In the future, the method’s generalizability can be further evaluated and enhanced by incorporating samples of dyed fabrics, establishing a dataset covering various dyeing conditions, and conducting validation across different colors and dye types. In addition, candidate discriminative intervals are derived from one internal validation split and training result. Their stability should be further assessed using repeated group splits, cross-batch validation, or bootstrap analysis. Molecular-vibration assignments for candidate intervals also require independent evidence from FTIR, Raman spectroscopy, or controlled-composition experiments. Future work should expand the range of sample sources and compositions and combine stability assessment with supplementary spectral experiments to improve the reliability of candidate discriminative bands and their molecular-vibration interpretations.

## Figures and Tables

**Figure 1:**
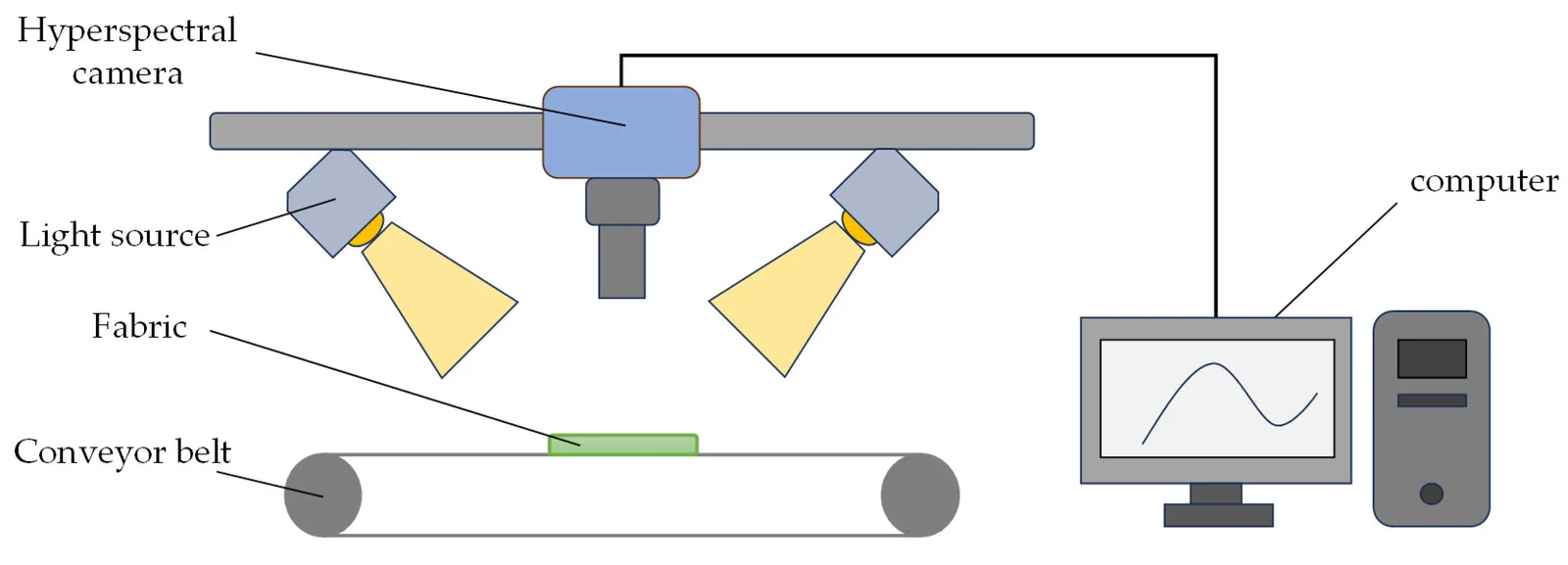
Near-infrared hyperspectral acquisition system.

**Figure 2:**
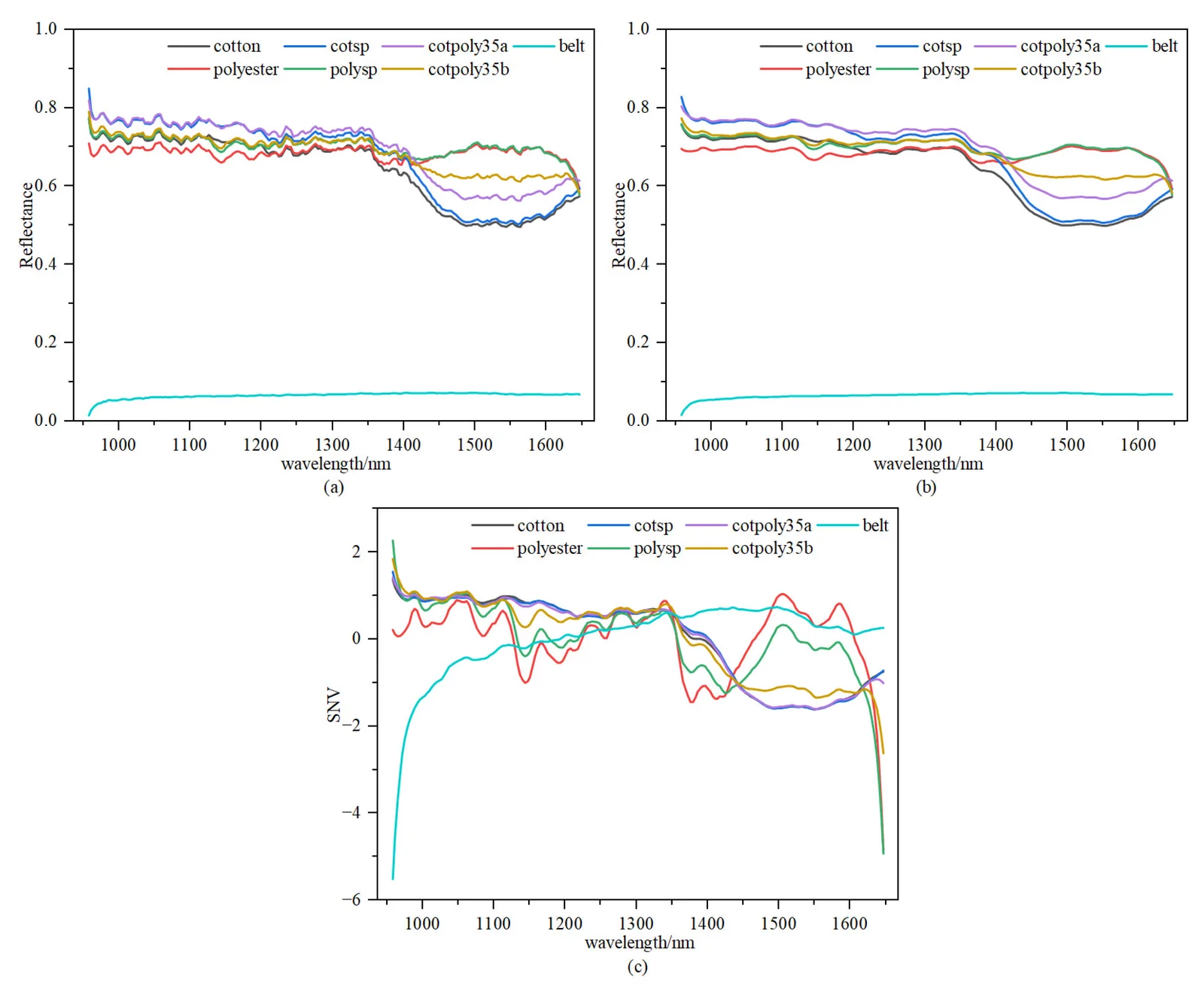
Mean spectra under different preprocessing methods. (**a**) Raw spectra; (**b**) SG-smoothed spectra; (**c**) SG-smoothed and SNV-corrected spectra.

**Figure 3:**
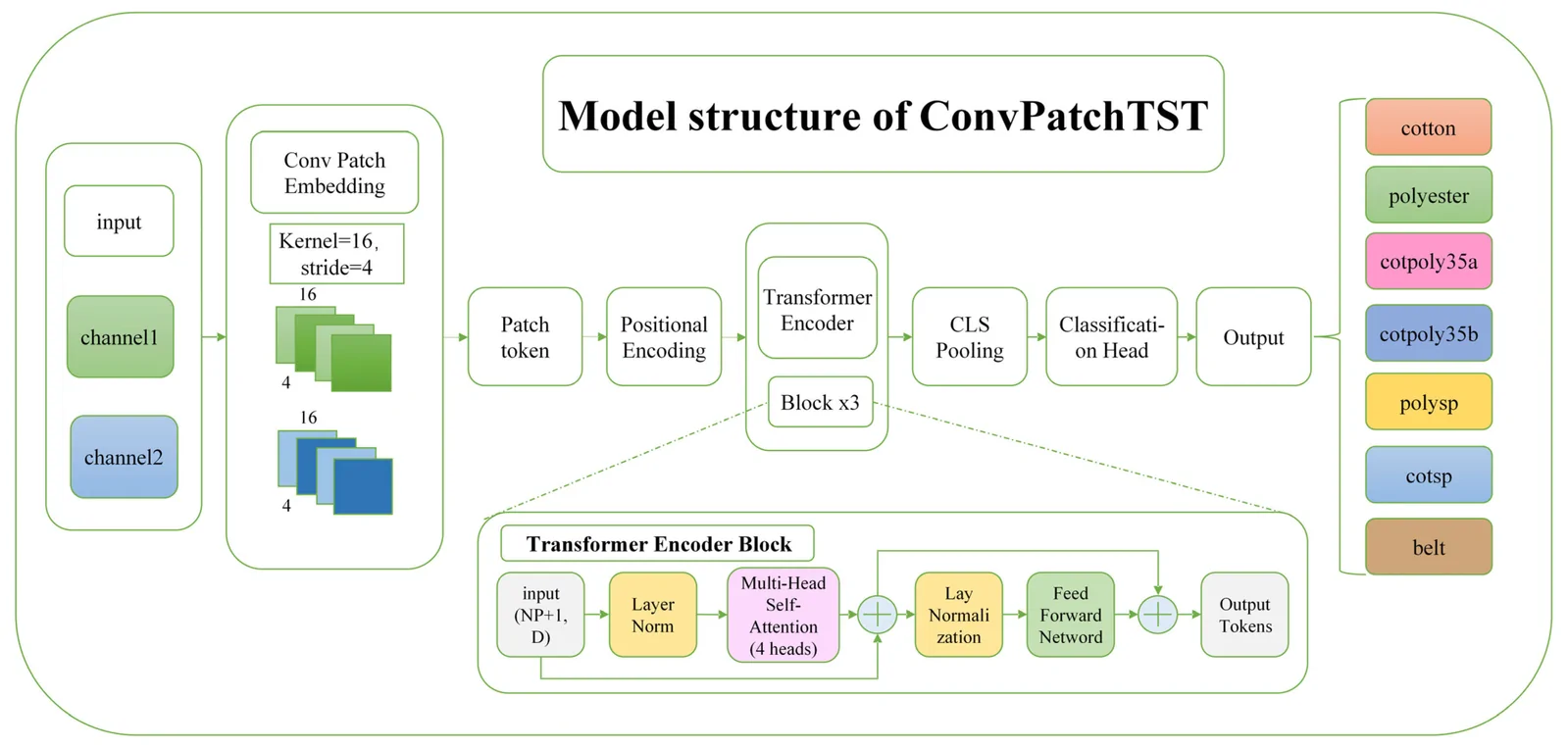
Architecture of ConvPatchTST.

**Figure 4:**
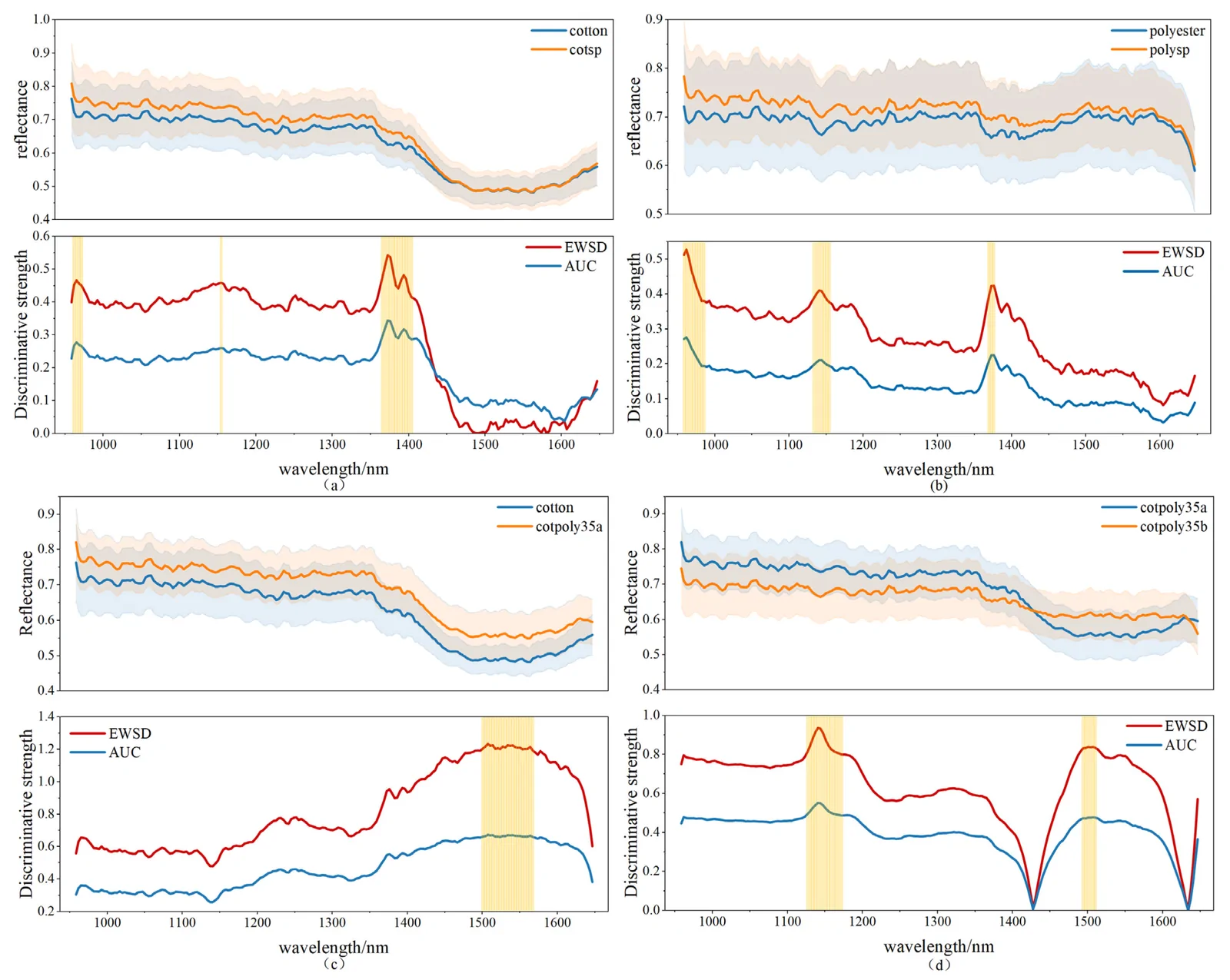
Spectral evidence for discriminating four pairs of similar materials. (**a**) cotton/cotsp; (**b**) polyester/polysp; (**c**) cotton/cotpoly35a; (**d**) cotpoly35a/cotpoly35b. The yellow highlighted regions indicate intervals that exhibit relatively high combined EWSD and AUC intensity and that appear continuously across adjacent bands.

**Figure 5:**
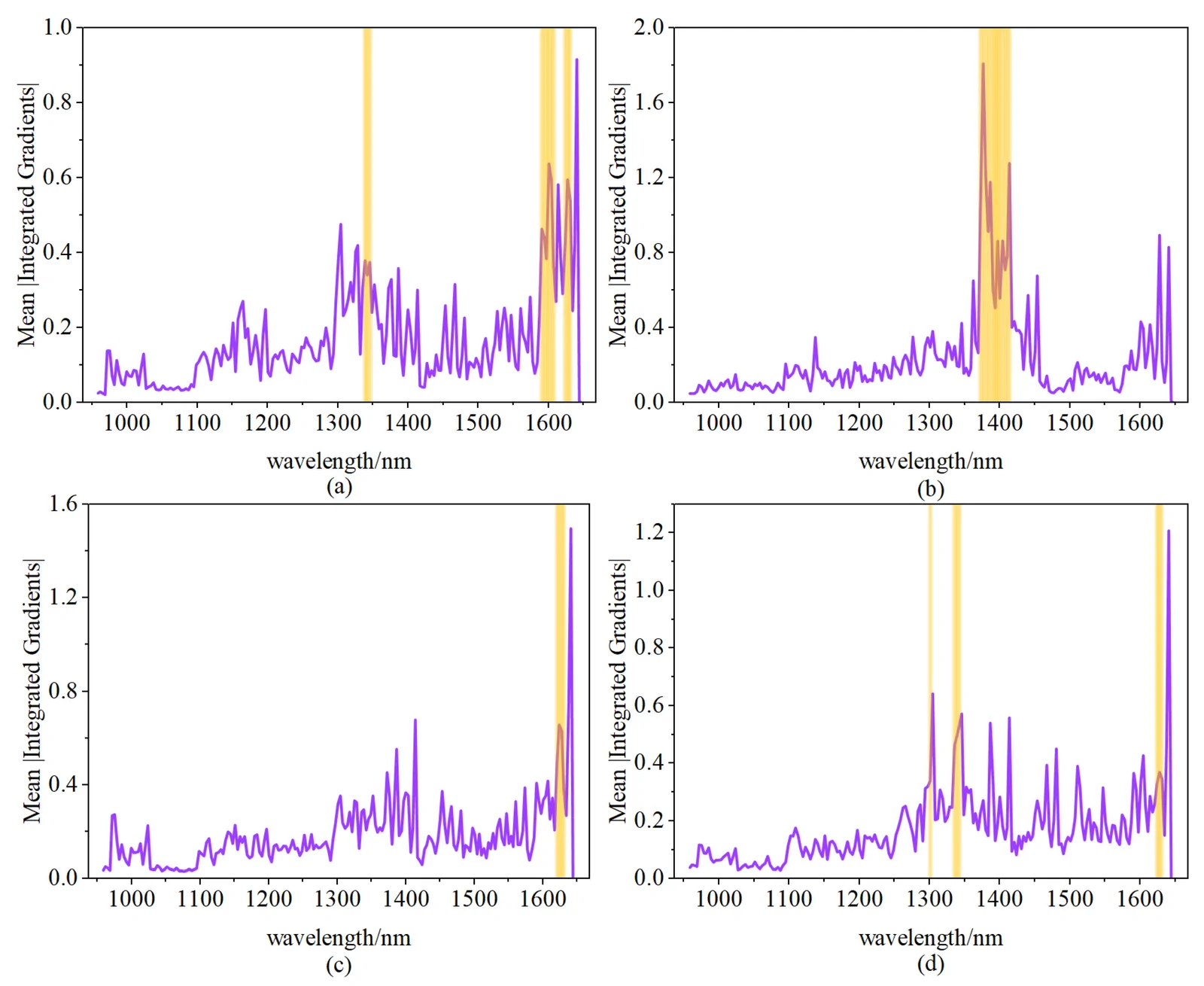
Band attribution results of ConvPatchTST. (**a**) cotton/cotsp; (**b**) polyester/polysp; (**c**) cotton/cotpoly35a; (**d**) cotpoly35a/cotpoly35b. The yellow highlighted intervals in the figure represent the highly attributed bands in the model’s discriminative basis.

**Figure 6:**
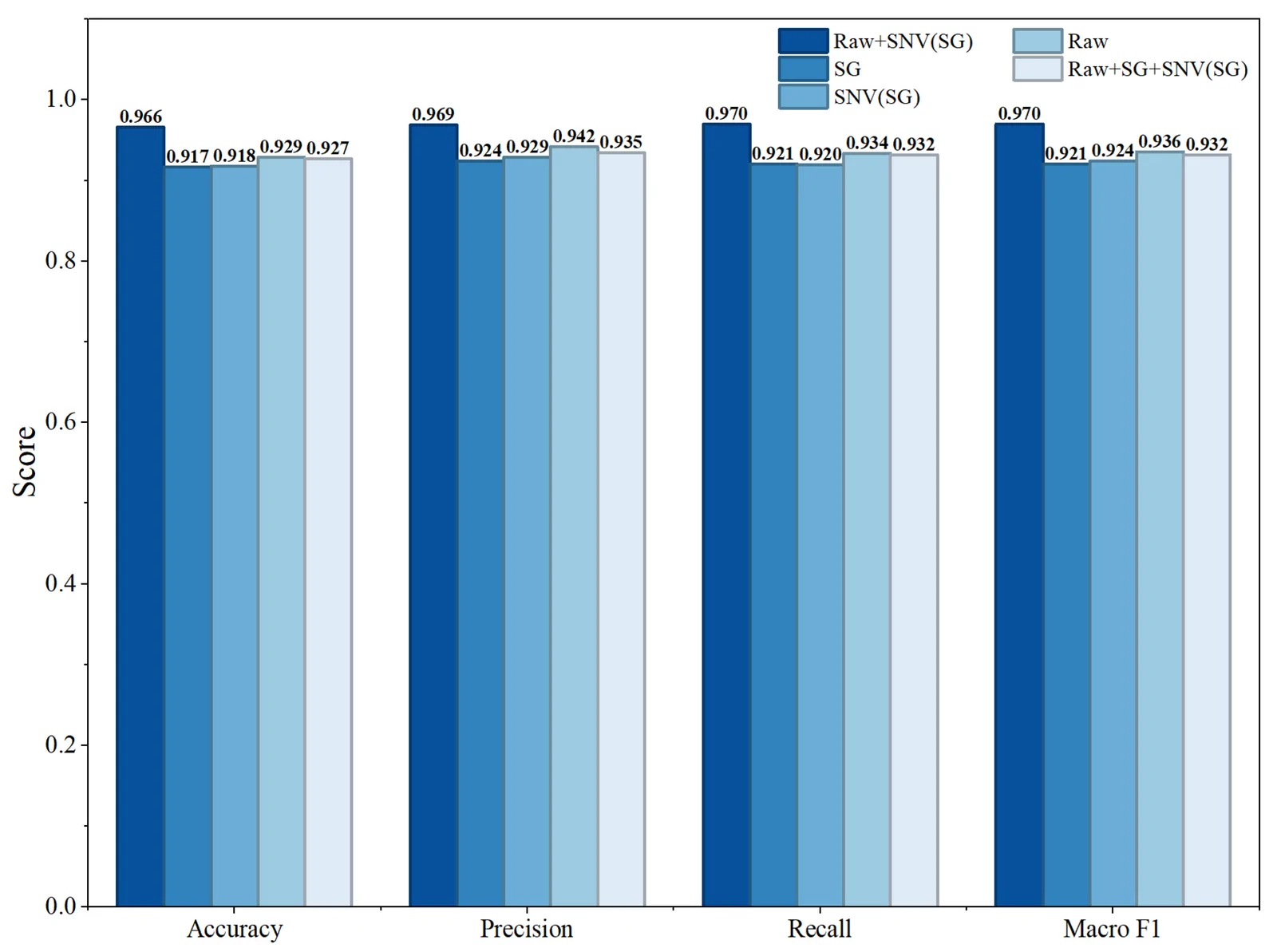
Classification performance for different channel inputs.

**Figure 7:**
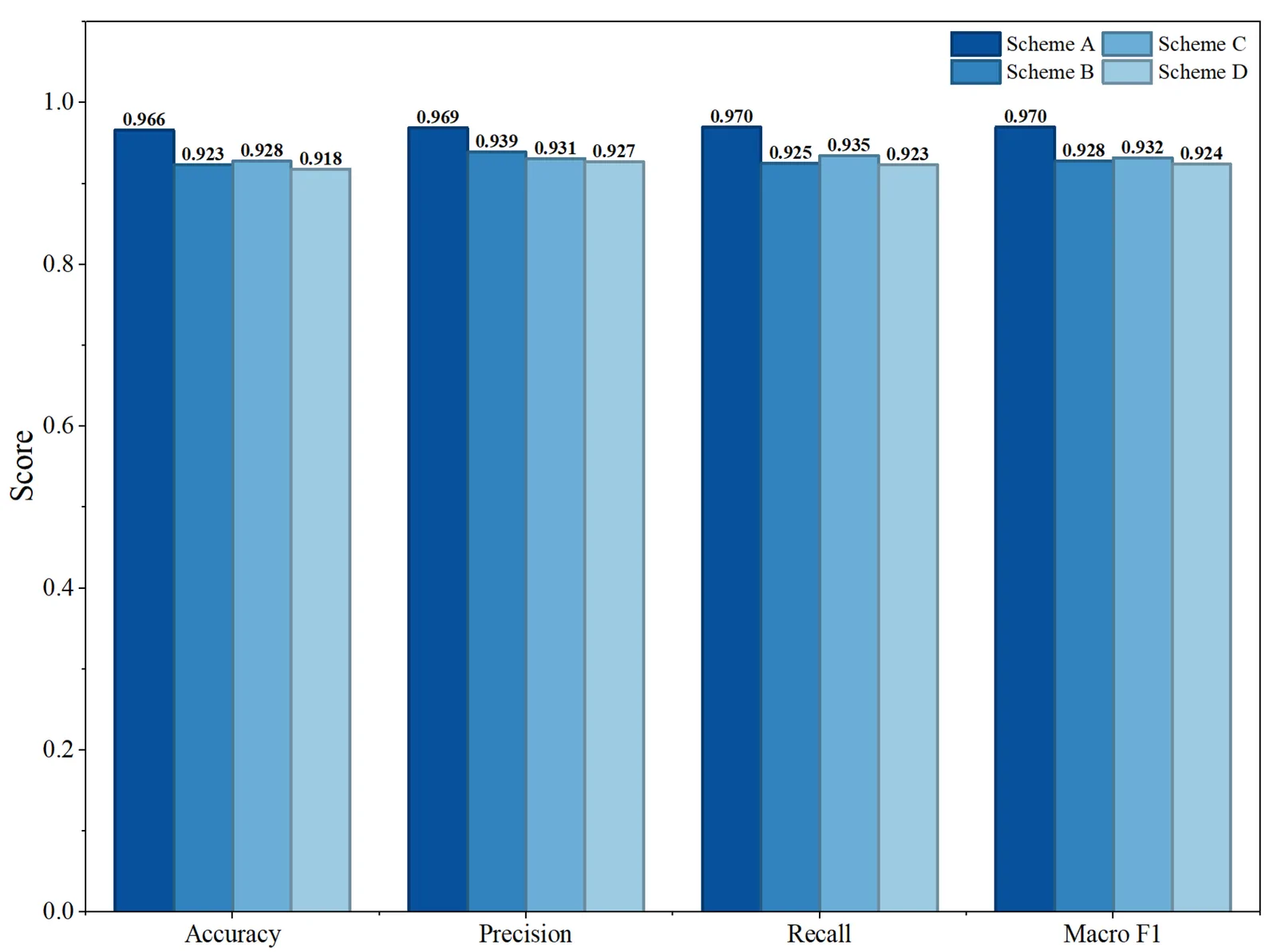
Classification performance for different dual-channel combinations.

**Figure 8:**
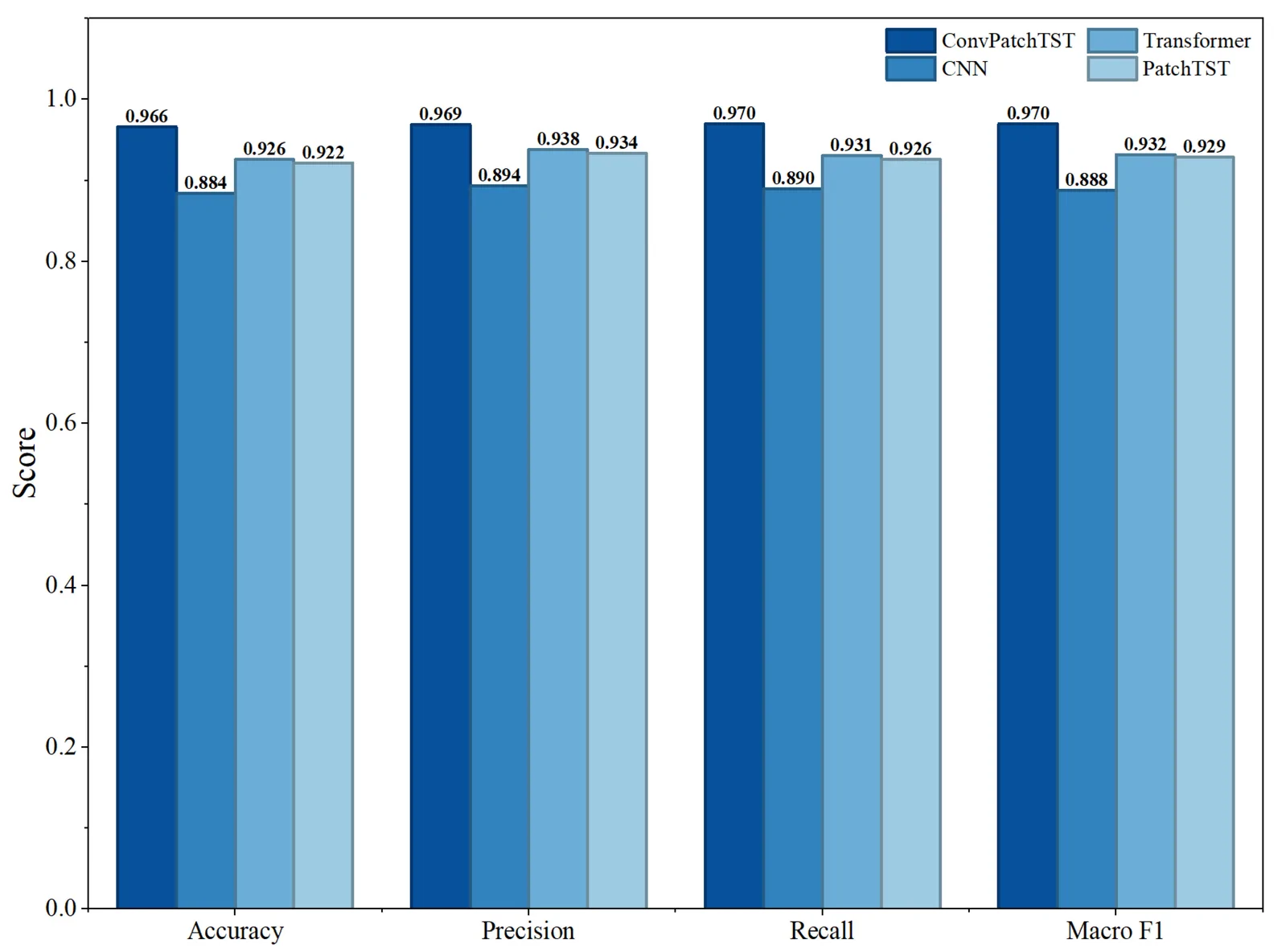
Classification performance of different models.

**Figure 9:**
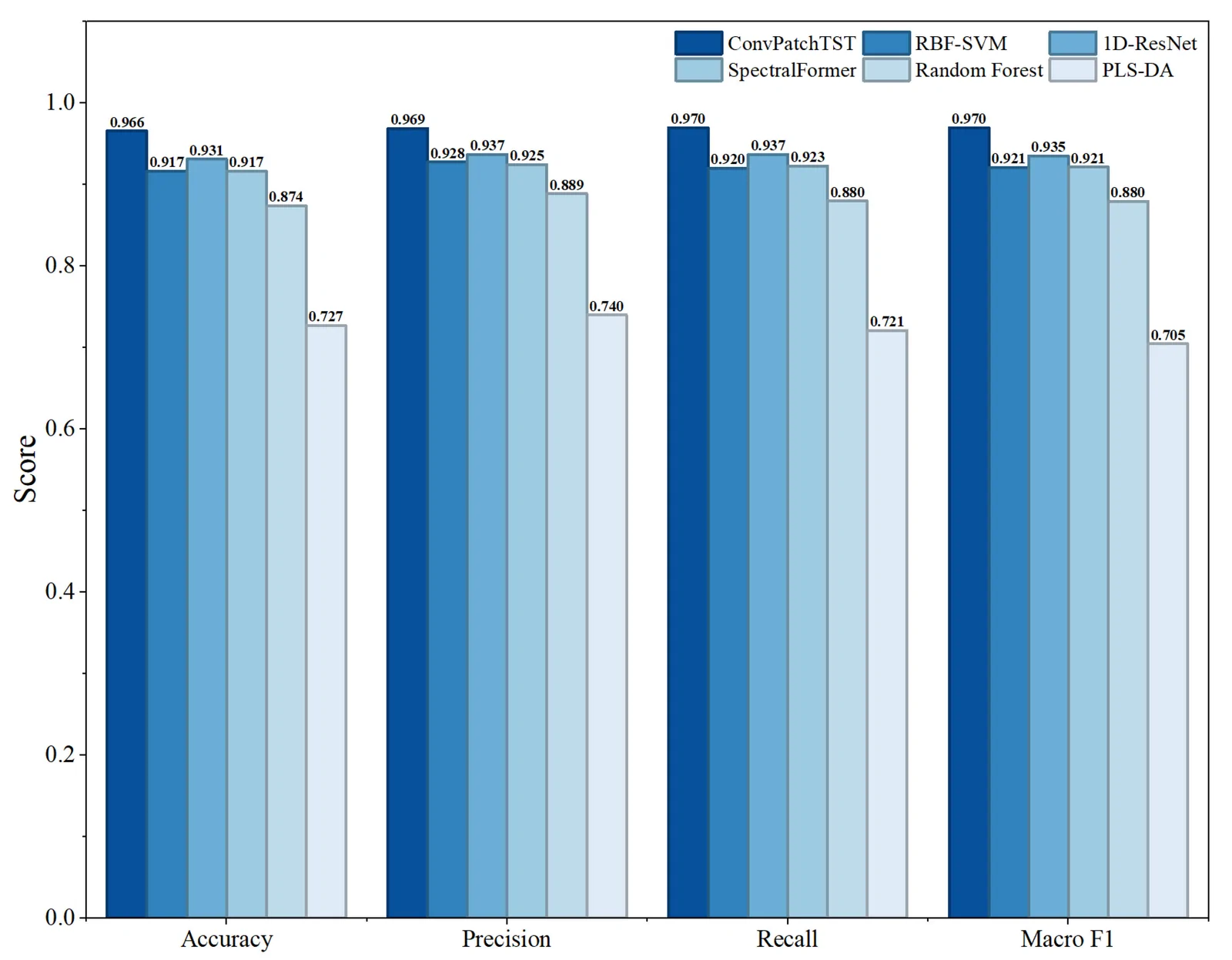
Overall classification performance of different models.

**Table 1:** Material composition and number of fabric samples.

Class	Fiber Composition	Fabric Samples
cotton	100% cotton	70
polyester	100% polyester	46
cotsp	cotton–spandex blend, with a polyester content ranging from 1% to 10%	48
polysp	polyester–spandex blend, with a polyester content ranging from 1% to 10%	73
cotpoly35a	cotton–polyester blend, cotton ≥ 35%	45
cotpoly35b	cotton–polyester blend, cotton < 35%	22

**Table 2:** ConvPatchTST model parameters.

Item	Setting
Optimizer	AdamW
Initial learning rate	8 × 10^−4^
Learning-rate scheduler	OneCycleLR
Maximum learning rate	4 × 10^−3^
Weight decay	2 × 10^−4^
Batch size	256
Maximum epochs	36
Minimum epochs	20
Loss function	Balanced Softmax
Transformer encoder layers	3
Attention heads	4
Hidden/embedding dimension	128
Feed-forward dimension	256
Random seed	43
Validation frequency	Every epoch
Early-stopping criterion	Best validation Macro F1; minimum 20 epochs; patience 4

**Table 3:** Numbers of training and validation samples.

Class	Training Set	Validation Set	Total	Training Swatches	Validation Swatches
cotton	41,671	10,565	52,236	55	15
polyester	29,600	7200	36,800	37	9
cotsp	30,376	7500	37,876	38	10
polysp	44,702	11,282	55,984	58	15
cotpoly35a	36,692	9173	45,865	36	9
cotpoly35b	37,280	9507	46,787	18	4

**Table 4:** Statistical discriminative intervals and model attribution intervals for pairwise materials.

Material Pair	Statistically Discriminative Intervals/nm	Mean EWSD	High-Attribution Model Intervals/nm	Mean Attribution
cotton/cotsp	1362.1–1407.1; 961.6–971.8; 1150.9–1157.9	0.469; 0.456; 0.457	1623.7–1630.6; 1590.6–1607.0; 1338.8–1345.4	0.519; 0.480; 0.364
polyester/polysp	958.5–985.5; 1369.0–1379.6; 1133.5–1154.4	0.449; 0.408; 0.395	1372.4–1413.6	0.945
cotton/cotpoly35a	1500.5–1567.0	1.214	1620.4–1630.6	0.541
cotpoly35a/cotpoly35b	1126.7–1175.8; 1493.9–1510.5	0.855; 0.836	1335.3–1345.4; 1297.3–1304.3; 1623.7–1630.6	0.515; 0.433; 0.348

**Table 5:** Classwise F1-scores for different channel inputs.

Class	Raw + SNV(SG)	Raw	SG	SNV(SG)	Raw + SG + SNV(SG)
cotton	0.945	0.918	0.943	0.957	0.940
polyester	0.996	0.974	0.936	0.952	0.957
cotsp	0.961	0.974	0.916	0.913	0.979
polysp	0.994	0.931	0.898	0.898	0.927
cotpoly35a	0.931	0.897	0.911	0.912	0.927
cotpoly35b	0.960	0.860	0.843	0.833	0.888
belt	1.000	1.000	1.000	0.998	1.000

**Table 6:** Dual-channel input combinations.

Scheme	Input Channel Combination	Design Rationale
Scheme A	Raw + SNV (SG)	Raw reflectance and stable spectral shape after smoothing and scatter correction
Scheme B	Raw + SNV (Raw)	Raw reflectance and spectra corrected directly with SNV
Scheme C	Raw + SG (Raw)	Raw reflectance and smoothed spectra
Scheme D	SG (Raw) + SNV (SG)	Two representations both derived after SG processing

**Table 7:** Classwise F1-scores for different dual-channel combinations.

Class	Scheme A	Scheme B	Scheme C	Scheme D
cotton	0.945	0.964	0.956	0.954
polyester	0.996	0.975	0.949	0.940
cotsp	0.961	0.923	0.935	0.939
polysp	0.994	0.917	0.889	0.880
cotpoly35a	0.931	0.897	0.942	0.925
cotpoly35b	0.960	0.820	0.856	0.828
belt	1.000	1.000	1.000	1.000

**Table 8:** Classwise F1-scores for different models.

Class	ConvPatchTST	CNN	Transformer	PatchTST
cotton	0.945	0.898	0.908	0.899
polyester	0.996	0.921	0.953	0.958
cotsp	0.961	0.823	0.957	0.947
polysp	0.994	0.894	0.926	0.927
cotpoly35a	0.931	0.870	0.911	0.890
cotpoly35b	0.960	0.813	0.870	0.878
belt	1.000	0.999	1.000	1.000

**Table 9:** Comparison of classwise F1-scores across models.

Class	ConvPatchTST	1D-ResNet	SpectralFormer	RBF-SVM	Random Forest	PLS-DA
cotton	0.945	0.973	0.938	0.939	0.886	0.679
polyester	0.996	0.967	0.949	0.970	0.943	0.849
cotsp	0.961	0.946	0.918	0.899	0.808	0.299
polysp	0.994	0.910	0.901	0.915	0.888	0.902
cotpoly35a	0.931	0.929	0.910	0.900	0.856	0.706
cotpoly35b	0.960	0.822	0.834	0.826	0.777	0.875
belt	1.000	1.000	1.000	1.000	1.000	0.996

## Data Availability

No new data were created or analyzed in this study. Data sharing is not applicable to this article.
